# “The Heart Desires but the Body Refuses”: Sexual Scripts, Older Men’s Perceptions of Sexuality, and Implications for Their Mental and Sexual Health

**DOI:** 10.1007/s11199-017-0822-3

**Published:** 2017-09-09

**Authors:** Sylivia Karen Rutagumirwa, Ajay Bailey

**Affiliations:** 1 Population Research Centre, Faculty of Spatial Sciences, University of Groningen, Groningen, The Netherlands; 2 Transdisciplinary Center of Qualitative Methods, Manipal University, Manipal, India

**Keywords:** Older men, Sexuality, Masculinity, script’s conformity, Sexual and mental health

## Abstract

We use sexual scripting theory in the present paper to gain a better understanding of older men’s perceptions of their sexuality in relation to dominant Tanzanian cultural norms of masculinity. Qualitative in-depth interviews were conducted with 15 older men, and ten focus group discussions were conducted with 60 men aged 60–82. Participants’ recruitment was guided by theoretical sampling strategies. Consistent with the principles of grounded theory, data collection and analysis occurred simultaneously. Our findings indicate that Jando (male initiation rites) serves as a script for male sexuality that outlines the expectations and rewards of male sexuality. Adhering to masculine sexual script affects older men’s perceptions of their sexuality in later life and has detrimental effects on their well-being. Older men were concerned with changes in their sexual life, such as the decline in their sexual performance. The majority of the participants said they felt emotionally distressed about the age-related decline in their body and in their sexuality, and they reported that their inability to conform to male sexual scripts undermined their sense of masculinity. Several of the participants reported that in an effort to regain their previous sexual performance, they had turned to remedies and strategies of questionable appropriateness and effectiveness. Our study suggests that older men may benefit from age-related interventions tailored to their cultural background. These interventions may require trained health care providers on mental health issues to bridge the gap between the internalized scripts of ideal male sexuality and the reality of aging.

**Electronic supplementary material:**

The online version of this article (10.1007/s11199-017-0822-3) contains supplementary material, which is available to authorized users.

The world’s population is aging rapidly, and although the aging of the population is a global phenomenon, this trend is progressing most rapidly in developing countries (United Nations Department of Economic and Social Affairs—UNDESA 2013). By 2050, nearly 80% of the world’s older population will be living in developing countries. The United Nations projects that between 2020 and 2050, the absolute number of Tanzanians over age 60 will almost triple, increasing from 2.95 million to 8.39 million (United Nations 2008). Thus, as populations age, issues affecting older people are becoming increasingly important.

The sexual health of older adults is among the core issues that demand special attention (Gott and Hinchliff 2003; Manjula and Beard 2013). In particular, sexual health of older people in Africa needs special attention due to the rollout of antiretroviral therapy (ART) in Africa which has increased the life expectancy of HIV-infected people (Gómez-Olivé et al. 2013; Negin and Cumming 2010; Negin et al. 2012). As the young people who have been infected with HIV/AIDS get older, the age groups impacted by this problem have been changing (Negin et al. 2012). As the burden of HIV infection shifts to older age groups, the risk of infection among older people may be expected to increase (Manjula and Beard 2013; Negin et al. 2012). However, in health systems in Africa, the sexual health of older people is often overlooked and neglected in interventions and policies (Freeman and Coast 2014; Manjula and Beard 2013; Negin et al. 2012). The primary aim of the present study is to use sexual scripting theory to examine older men’s perceptions of sexuality in relation to the norms of masculinity (i.e., men’s sexual scripts). We argue that a thorough understanding of older men’s perceptions of sexuality, as embedded in the socio-cultural norms of masculinity, is crucial for designing age- and gender-sensitive sexual health interventions that address their sexual health needs.

## Sexual Health and Sexuality of Older People in Africa

Gendered norms around sexuality affect how sexual behaviour and sexual health issues are reported (UNFPA 2014). Although the sexual health needs of young men have been placed squarely on research and policy agendas, there are still many unanswered questions about issues related to sexual health of older men. The sexual health and the sexual well-being of older people in Africa are often overlooked and neglected in interventions and policies due to various reasons including the lack of data on the sexual health needs of older people (Freeman and Coast 2014; Gutsa 2011). Efforts to collect sexual health data in Africa are primarily focused on issues surrounding fertility and chronological age (Freeman and Coast 2014). In addition, in many African cultures, including in Tanzania, the subject of sexuality among older people remains largely taboo (Nyanzi 2011; Okiria 2014), and there is a general discomfort with the notion that an older person may be interested in sex. For example, studies conducted in Uganda have shown that sexual activity among older widows is heavily stigmatized because it is considered shameful for aging women to be sexually active because their sexual behaviours are not procreative (Nyanzi 2011; Okiria 2014). Furthermore, in his in-depth ethnographic study in Ghana, Van der Geest (2001) identified a considerable amount of ambivalence toward sex during old age: Whereas sex was perceived as an activity in which older adults should no longer be interested, engaging in sex tends to confirm the vitality and the status of older adults.

Further reasons for exclusion of older people from sexual health programs are based on assumptions that are embedded in the biomedical and epidemiological research that informs sexual health interventions (Knodel 2006; Tietz 2010). Most of the biomedical and epidemiological interventions that gained momentum after the HIV/AIDS epidemic are youth-focused. Hence, older people are routinely excluded from HIV-screening programs, and safe sex interventions almost exclusively target younger people (Freeman and Anglewicz 2012; Nkya et al. 2006). Nevertheless, empirical evidence increasingly suggests that sexual interest is not significantly affected by aging (Gott and Hinchliff 2003). For instance, Gutsa’s (2011) ethnographic study in Dzivaresekwa Zimbabwe revealed that even though older adults were often sexually active, they were wrongly regarded by healthcare providers as being sexually inactive, and thus as not susceptible to contracting sexually transmitted infections (King et al. 2010).

Freeman and Anglewicz (2012) is one of the few studies on sexual health in Africa that focused on older people. Their comparison of the sexual behaviour and HIV-infection patterns among 3719 individuals aged 15–49 with those of individuals aged 50–64 and 65+ in Malawi indicated that among the 65+ age group, levels of sexual activity and HIV infection were much higher among men (73.8%) than among women (26.7%). Similarly, recent analyses of 750 patients aged 50+ in Uganda showed that 40% remained sexually active after their HIV diagnosis (Funk et al. 2012; Negin et al. 2012). These findings further indicated that older patients had a significantly higher rate of STIs than younger patients did. In the present paper, we argue that “it is not possible to achieve one’s sexual health without consideration or expression of one’s sexuality, which underlies much behaviour associated with sexual health” (UNFPA, 2014, p, 14). Thus gaining information about the sexuality of older men can contribute significantly to sexual health interventions aimed at older people. Such interventions are needed to ensure that older men are able to make informed decisions about their health and sexuality (UNFPA 2014; World Health Organization [WHO] 2010).

## Sexual Script Theory

Sexual scripts are the approved norms regarding sexuality that individuals embrace, internalize, and endorse through a process of socialization (Simon and Gagnon 1986). Fundamental to sexual script theory is the notion that sexual scripts are inherently embedded in a cultural context in which cultural norms provide guidelines for appropriate behaviours, emotions, and cognitions for men and women in sexual experiences (Simon and Gagnon 1986). Scripts become the models people use to interpret and respond to sexual situations (Rose and Frieze 1993; Simon and Gagnon 1986). Arguably people draw upon these scripts when judging their sexual experiences and when engaging in sexual behaviours (Stephens and Eaton 2014, p.2). A basic premise of sexual script theory—and one that is especially relevant for our study—is that sexuality is learned from culturally available messages that set guidelines regarding sexual behaviour and activities (Frith and Kitzinger 2001).

Research specific to sexuality and scripting has revealed the influence of differing cultural values on notions of masculinity and their impact on sexual health (Maticka-Tyndale et al. 2005; Stephens and Eaton 2014). For example, in Western cultures, sexual scripts depict men as sexually active, ready for sex and expressing sexual dominance over women (Courtenay 2000; Stephens and Eaton 2014). In contrast, in a number of African cultures, sexual scripts are centred around the discourse of coercion and aggression in sexual relationships (MacPhail and Campbell 2001; Maticka-Tyndale et al. 2005). Arguably, aggression in sexual interactions is intertwined with and interchangeable from social and biological pressure. Thus, men are expected to be sexually aggressive, and women are expected to be submissive and compliant (Maticka-Tyndale et al. 2005).

Moreover, the literature has shown that the details of sexual scripts are acquired during childhood and adolescence (Rose and Frieze 1993; Wiederman 2015). In traditional African cultures, the scripting of sexuality includes initiation rites performed during adolescence which includes formal instruction in sexual issues and gender matters (Maticka-Tyndale et al. 2005). In Tanzania these initiation rites includes the Jando (male initiation rites) and Unyago (female initiation rites)*.* These initiation rites institute strict social and physical control measures, especially for the sexual behaviour (Abeid et al. 2014; Maticka-Tyndale et al. 2005). For instance, Jando provides a cultural script that regulates men’s sexuality, plays a fundamental role in shaping young boys’ transition to sexual maturity, and communicates to them about their expected sexual roles (Abeid et al. 2014; Tumbo-Masambo 2004). Thus, beyond male circumcision, the core function of Jando is to inculcate the values of a culture; it provides a cultural script that regulates male sexuality and gender roles so that sexual identity and gender identity are enshrined through Jando (Ntukula 1994; Tumbo-Masambo 2004).

In the course of Jando initiation, young men receive specific messages regarding sexual normalcy, sexual power, and the appropriate forms of sexual expression for a man. In short, the scripting of sexuality (through Jando) is intended to socialize and instruct young men in life skills as well as sexual and gender matters (Abeid et al. 2014; Maticka-Tyndale et al. 2005). It is therefore anticipated that, once internalize, the initiates will use the script to interpret and respond to sexual situations (Simon and Gagnon 1986; Tumbo-Masambo 2004). Arguably, the knowledge and the skills that are transmitted through Jando are internalized and lay a foundation for a man’s sexual life (Abeid et al. 2014; Tumbo-Masambo 2004). However, previous empirical research has not investigated the question of whether the sexual scripts acquired by men early in life (in Jando) are reflected in their perceptions, and particularly in the meaning they assign to their sexuality later in life.

## Sexuality and Masculinity in Later Life

A major concern of feminist gerontologists is to bring together social and cultural dimensions in the study of aging (Sandberg 2013). One of the most remarkable contributions in the field is from Gullette (2004) who asserted that rather than being driven by biological processes of aging, we are just as much aged by culture. Arguably, the ways in which older men respond to sexual changes, and how they integrate these changes into their sense of well-being, are embedded in cultural frameworks (Minichiello et al. 2005). Feminist gerontologists and sexuality researchers have been slow to incorporate sexual script theory into the study of masculinity and sexuality in later life. Instead, the empirical work that has been guided by the scripting approach has largely been conducted by feminists and sexuality researchers who have used the approach as a framework for studying and analyzing the sexuality of young people.

For instance, in Kenya, Maticka-Tyndale et al. (2005) used scripting theory to develop an in-depth understanding of how sexuality is experienced by Kenyan young people and examine the socio-cultural contexts in which it is embedded. They found that sexual encounters were described as both mundane and inevitable as well as followed a predetermined scripted sequence of events and interactions in which young women and men played complementary roles (Maticka-Tyndale et al. 2005). Similarly, Izugbara and Undie (2008) used sexual script theory to investigate contextually rich talk about sex and sexual activity among Malawian male youth. They found that internalized sexual scripts were the main driver of youth’s vulnerability (i.e., prompted them into risky practices) because these scripts constitute masculinity as very fragile and in need of constant protection, making these male adolescents wary both of female partners who refused them sex and of sexual practices which offered little or no control and power over women (i.e., that raised suspicions about their manliness). Sexual script theorists such as Simon and Gagnon (1986) argued that the sexual scripts are age-appropriate/graded and that the kinds of sexual behaviour that are considered acceptable differ depending on whether people are younger or older. However, none of known studies on sexuality and scripting in Africa go beyond younger generations and beyond describing HIV risk-related behaviours. Hence, our study focused on sexual scripting of older men in relation to norms of masculinity.

## Method

### Participants

A qualitative study was carried out among older men, aged 60 to 82 and who live in the Coast Region of Tanzania (Pwani). We used a qualitative research design because the topic of sexuality had not previously been examined among older people in Tanzania and because a qualitative design is well suited to the collection of data on sensitive topics. The study was conducted from November 2012 to June 2013, and we obtained approval from the relevant ethics committees. Purposive and snowball sampling strategies were used to recruit potential participants for our study—participant recruitment was guided by theoretical sampling. Inclusion criteria included being born and raised in Pwani, living in Pwani currently, and being a man aged 60+. A total of 75 participants participated in our study, of whom 60 took part in focus groups and 15 completed in-depth interviews. Participants were recruited at one time with no overlap between focus group participants and in-depth interviewees.

The 60 participants who participated in our focus groups ranged in age from 60 to 82 years-old, with 24 (40%) aged 60–69, 24 (40%) aged 70–79, and 12 (20%) 80 or older. Most (54, 90%) were currently married, with 6 (10%) being widowed, divorced, or single. Participants were virtually split between being monogamous (29, 48%) and being polygamous (31, 52%), as well as between living in urban (30, 50%0 and rural (30, 50%) areas. Their level of education spanned a spectrum from having no formal education (18, 30%) through primary education (22, 37%) and secondary education or vocation skills (14, 23%) to completing college/university (6, 10%).

The 15 participants whom we interviewed individually were aged between 60 and 82. Their marital statuses were correspondingly diverse, ranging from married with more than one wife (polygamy), married with one wife (monogamy), and single/widower. Typically the men’s wives were younger than they were at the time of interview. The biggest age gap between participants and their spouse was 20 years and the smallest age gap was 5 years. Generally, older men had experienced a minimal amount of formal education, and a good number of them attended religious education (madras).

### Procedure

Because ours is an interpretive study that emphasizes people’s perceptions of meaning, we used the method of grounded theory to enable identification of the issues from participants’ own perspectives, that is, getting the emic perspectives from the people themselves, to reveal their perceptions about sexuality. We therefore asked research questions that were open. Some of our initial research questions were: What are the norms/scripts of masculinity in Tanzania?, How do norms/scripts for male sexuality shape the ways in which older men perceive their sexuality in later life?, and How do masculine sexual scripts play into older men’s perceptions of aging-related change in body functioning? Questions were open and became more refined with the evolving analysis (Corbin and Strauss 2008). Data were collected using focus group discussions (FGDs) and in-depth interviews (IDIs). During the pilot phase, the authors tested the applicability of the questions and whether their approach to holding a group discussion and interviews were effective. (The complete guides we used for both the focus groups and individual interviews are available as an online supplement.)

#### Focus Group Discussions

Focus group participants were purposively recruited by the first author with the help from village executive officers, ward executive officers, and leaders of organizations for older adults. These focus groups were convened to explore the shared norms of masculinity and male sexuality. The study also adopted focus group techniques to identify an initial set of themes, specifically with a view to guiding the individual interviews with older men. In total ten focus group discussions were conducted with 60 older men. Focus groups were also used to gain a general overview of the vocabulary older men use in relation to sexuality. The focus group guide was based on the literature; however as the study proceeded and we established better insiders’ views, we adjusted our research questions in order to accommodate certain (emic) constructs and categories that emerged. The guide for our focus groups was also developed through feedback from the pilot study conducted by the first author in September 2012. Some of the focus group questions included: What are the traditional masculine norms/scripts in your community? and How do Jando influence the ways in which older men perceive on their masculinity and sexualities?

Each focus group discussion involved six participants and lasted between approximately 90 and 125 min. The FGDs were held in locations the participants chose. Participants were grouped based on social identities such as age (60–69, 70–79, and 80+) and marital status (married-monogamy, married-polygamy, widowed or divorced/single). Assigning participants to groups with similar characteristics removed social norms and hierarchies that could create barriers to open discussion. This approach increased the likelihood that participants would feel comfortable with each other and would therefore contribute openly to the discussion. Grouping was also done on the basis of location. Thus, many of our focus group participants knew each other because the majority came from the same community or neighborhood.

All of the discussions were conducted in Kiswahili and were led by an experienced FGD moderator (the first author) assisted by two qualified qualitative male researchers. All of the FGDs were audio-taped with the participants’ consent and were immediately transcribed and translated from Kiswahili into English. The small number of group participants (six per group) allowed the moderators to maintain a high level of involvement with the groups and to explore the research topics in the expected depth. The moderator intervened only occasionally during the discussion, mainly to remind the participants of the theme being discussed and to introduce new themes. At the end of each question we made sure that the common opinion had been expressed and agreed upon by all the participants in the discussion. At the conclusion, the moderator summarized the main points covered and asked the participants to verify that the information provided was an accurate summary of the discussion. Generally, views, opinions, and issues generated from the FGDs were used to fine-tune and polish the guides that were used in the in-depth exploration of the aspects at the individual level. Thus, the data from FGDs and IDIs were mutually informative (Lambert and Loiselle 2008).

#### In-Depth Interviews

After conducting the focus group and gaining insights on the norms of masculinity and male sexuality, the in-depth interview guide was developed to gather deeper information about individuals’ perceptions of sexuality in relation to masculinity norms. Participants for IDIs were recruited through snowballing technique, whereby the participants (from FGD participants) were asked to facilitate the initial recruitment effort by recommending others who fit the criteria for participation—then the initial IDIs participants recommended others participants. In total, 15 in-depth interviews (IDIs) were conducted among older men. The interview guide contained open-ended questions that were primarily guided by the findings from the focus group (i.e., questions from FGDs were re-worded to reflect individual’s experiences). In a nutshell, FGDs guided the exploration of individual accounts, whereas subsequent individual data further enriched the conceptualisation of phenomenon (Lambert and Loiselle 2008), as well as revealed themes that were not in FGDs such as aging body and sexuality.

The first author, assisted by a qualified qualitative male researcher, conducted the in-depth interviews at a place and a time selected by the participant. The length of interviews ranged from 1 to 2 h. Given that the interview topic was considered to be sensitive, some opening questions were designed to establish a rapport and to give participants an opportunity to direct the research discussion. Interview questions and additional prompts were developed to clarify issues raised by these questions. Each interview started with the open-ended question such as: What is expected of you as a man?” An example interview question is: What are individual’s perceptions of the impact of body-related changes in sexual functioning. Commonly participants compared and contrasted their current perceptions of “masculine sexuality” with that of their younger selves. In line with grounded-theory methods, the salience of such comparisons was investigated.

Similar to the FGDs, all the interviews were conducted in Kiswahili, were audio-taped, and were immediately transcribed and translated from Kiswahili into English. After each interview, the researchers read the transcripts and wrote detailed memos about the emergent perspectives and themes identified in the interviews. These outcomes were discussed with a broader research team, and the issues that were raised were explored further in subsequent interviews with other participants. The interview guide evolved through a process of data collection that allowed us to focus on concepts and themes as they emerged. We continued to collect and analyze data by interviewing new participants until until data saturation was reached (Corbin and Strauss 2008).

### Research Positionality and Reflexivity

In qualitative research, a researcher is an instrument in the collection of data. Therefore, it is crucial to address the positionality of the interviewers in order to understand their potential impact. In our study, the interviews and the focus group discussions were conducted by the first author assisted by two men (trained qualitative researchers)*.* The first author’s social positioning (as a Tanzanian woman, sociologist, and a feminist fluent in Kiswahili) was an advantage because it enabled access to potential participants. On the other hand, being a relatively young woman compared to participants who were older men potentially served as a disadvantage. For instance, in the initial group discussions the first author realized that she was missing the vocabulary/euphemisms used by older men to refer to sexual relations; however, after a few sessions she was able to master the vocabulary. Additionally, this disadvantage was countered by the presence of two research assistants who shared demographic characteristics with the majority of the participants, including ethnicity, age, and sex. Furthermore, it is clear that our professional (sociologists, feminists, anthropologists and demographer) underpinned our research process at every level. Our professional background may have motivated some participants to take part in the study, perhaps due to the opportunity to speak about their sexual problems and get attention. The memo below, which the first author wrote during the data collection process describes the positionality of the researcher:

Memo: [2013-02-26 08:36:34]. Before I started data collection I struggled to imagine what my relationship to the participants would be. I asked myself whether they would be willing to open up to me. …I realized that, in order to be trusted by the participants, the personal characteristics of the interviewers, such as sense of humor, dress, and conduct would be more important in establishing rapport with and gaining the confidence of the participants than the age or sex of interviewer.

All in all, conducting research with older people demonstrated the fluid nature of identities—it seems that in Tanzanian cultural context and other social differentiations (positionality) override age and sex. Throughout the duration of the data collection the author maintained a reflexive journaling where she documented her feelings, perceptions, emotions as well as inquiries that guided the collection of data.

### Validity

All of the interview guides were initially written in English and then translated into Kiswahili by the first author, who is fluent in both Kiswahili and English. The guides were then checked by the second author, who is fluent in English. The back translations were done by an English-Kiswahili language editor with expertise in both languages and knowledge of gender issues. The guides were piloted by the first author. Changes were made to the questions the participants found difficult to understand. Subsequently, another back translation was done by an editor who is a native English speaker. We transliterated the passages that could not be easily translated into English. We did a back-translation to check items that might produce unexpected results. These tests confirmed that the translation was valid.

### Analyses

The purpose of the current study was to examine older men’s perceptions of sexuality in relation to norms of masculinity. We conducted data analysis, as guided by Corbin and Strauss (2008), with an ideal schedule of analyzing each interview prior to the next interview. Data analysis started with the first interview. Memos were used during the data collection phase, during the earliest data analysis phase, and later while performing data analysis. Although codes were largely inductive, the initial research guides were influenced by literature (designing of questions). As the study proceeded we established better insiders’ views, and we adjusted research questions in order to accommodate certain (emic) constructs and categories that emerge. Although we utilized grounded theory, we adopted the analytic cycle in which “analysis of qualitative data for theory development is an interaction between existing deductively derived theory and inductively derived empirical theory” (Hennink et al. 2011). Before beginning the coding process, we actively read the entire dataset—a process that Braun and Clarke (2006, p. 16) referred to as “immersion in the data.” The first author then developed a codebook (three FGDs transcripts and three IDI transcripts were sampled to develop the initial code book), and coded the data using the codebook. Additional codes were added as needed.

The steps of data analysis can be summarized as follows. Data collection and analysis were concurrent (e.g., analysis began with the first group discussion) and theoretical sampling permitted deeper, richer data on emerging themes during subsequent interviews. This process of simultaneous data collection and analysis allows for constant comparison, which compares different pieces of data for similarities and differences and identifies dimensions specific to categories/themes (Corbin and Strauss 2008). For the initial coding, we coded each transcript line-by-line, using participants’ language as label coding. The line-by-line coding process enabled us to stay as close as possible to the data, as well as to remain open to any theoretical concepts and categories that emerged (Corbin and Strauss 2008). At this stage, we generated a long list of codes, and used Atla.ti 7 to manage the coding process. Four transcripts were coded by two independent qualitative researchers to see the influence of the first author’s position on the coding. The first author then discussed these outcomes with the second author, and agreement was reached, before continuing with the analysis.

Once the initial coding was completed and upon agreement, we grouped codes into related categories—axial coding. Axial coding started by crosscutting or grouping codes into larger categories with the purpose of reassembling data from the open coding process (Corbin and Strauss 2008). For example the initial (open) codes were grouped into categories labeled as: Jando as a script for male sexuality; Jando a model for masculinity; Aging body; The loss of sexual performance; Fear and shame about old-age sexuality; Anxiety about old-age sexuality; Persistence of sexual desire in an aging body; Confusion regarding the persistence of sexual desire in an aging body; Silencing norms associated with old-age sexuality; and Norms of male sexuality and implication on sexual health*.* We stopped coding and categorizing data when we reached saturation (Charmaz 2006). The validity of the study was further enriched by analyzing memos. In the later stage, theoretical sampling was used to explore and test these emergent themes (Corbin and Strauss 2008). At that point, five core themes that represent the underlying meaning found in the categories emerged—themes that revealed older men’s perceptions of sexuality in relation to the norms of masculinity.

## Results

More detailed information about each participant interviewed and quoted here can be found in Table 1. The analysis of the participants’ accounts produced five interconnected themes (see Table 2): (1) Jando as a model/script for male sexuality and masculinity; (2) The aging body and the loss of sexual performance; (3) Fear, shame, and anxiety about sexuality in old age; (4) Confusion regarding the persistence of sexual desire in an aging body; and (5) The silencing norms surrounding sexuality at older ages and the implications of these norms for sexual health.

**Table 1:** Characteristics of participants who were interviewed

Pseudonym	Age	Location	Level of education	Occupation(includes those retired)	Marital status	Number of wives	The age of the wife/wives
MzeeAli	70	Urban	Primary education	Small business man	Married	2 wives	1st wife (52) 2nd wife (59)
MzeeAmani	74	Urban	Secondary education	Retired unskilled manual	Married	1 wife	60
MzeeAyubu	68	Urban	Advanced secondary education	Retired professional middle level	Married	1 wife	54
MzeeBabu	68	Urban	Primary education	Other non-manual	Widower	No wife	None
MzeeBwaki	78	Rural	No formal education/ Attended Madrasa	Farmer	Married	2 wives	1st wife (66) 2nd wife (52)
MzeeChande	74	Rural	Primary education	Fisher	Married	2 wives	1st wife (67) 2nd wife (45)
MzeeChilingi	69	Rural	No formal education/ Attended Madrasa	Farmer/fisher	Married	4 wives	1st wife (59) 2nd wife (54) 3rd wife (46) 4th wife (38)
MzeeKimbau	74	Urban	Primary education	Retired unskilled manual	Married	2	1st wife (69) 2nd wife (48)
MzeeKyondo	71	Urban	Graduate	Retired/ professional higher managerial	Married	1	66
MzeeMagari	72	Rural	No formal education	Farmer	Married	1 wife	62
MzeeMbwiga	75	Urban	Primary education	Former businessman	Married	Divorced 3 remained with first wife	1st wife (70) (remained)
MzeeMdundo	72	Rural	Primary education	Farmer	Married	1 wife	44
MzeeMutoka	80	Urban	Secondary education	Retired professional lower level	Widower	No wife	None
MzeeNassoro	78	Rural	No formal education/ Attended Madrasa	Farmer	Married	2	1st wife (70) 2nd wife (55)
MzeeOmary	71	Rural	Vocation skills	Unskilled manual/farmer	Married	3 wives	1st wife (66) 2nd wife (62) 3rd wife (43)

(Mzee) is a standard abbreviation for an older man’s title following immediately before proper name. Madrasa refer to religious education

**Table 2:** Themes, descriptions, coding, and examples

Theme	Description	Indicative codes	Example (direct quotes)
Jando (initiation rites) as a model/script for male sexuality and masculinity	Participants described Jando as a primary avenue through which they learned masculine norms in relation to sexuality. Jando serves a double purpose: as a gender script and as a sexual script.	• Jando as a source of sexual messages • Internalization of the sexual script • Jando as a script for male sexuality • Determining sexual experiences • Enforcement of the sexual script	“…A lot of teaching happens there (in Jando)… there is no way a young man could get such deep information about life skills, gender roles, and sex roles without passing through Jando training… They taught us how to be men, about our roles …the instruction on sexual matters was a lot (laugh)… yes, because that is the core of manhood.” (FGD^a^)
Aging body and loss of sexual performance	Participants reported the loss of their past sexual self and their inability to meet the sexual expectations they learned from Jando due to age-related changes in their body.	• Failing body • Loss of erectile functioning • Loss of masculine identity • The loss of the past sexual self • Unable to perform well sexually • Embodied changes	“…Aging is a painful thing for a man … when a man becomes old all the energy leaves him …the body refuses to respond to desires… the two of you (husband and wife) just stare at each other, the relationship changes… you start treating your wife as your sister! Ehe! A week passes, and another... even a month can pass without doing it (sex), you are afraid of trying, you may perform poorly…Eeh the body may betray you again…as a man you feel worthless… You fail again! …This is very stressful for a man… Your partner may think, ‘Maybe my husband is tired of me or he is running around’… The quarrel starts.” (MzeeMagari)
Fear, shame, and anxiety about old-age sexuality	Participants expressed fear/frustration related to their inability to fulfil sexual expectations and to their perception that they were no longer respected and had lost power and control over women. Their sexual decline thus caused them distress and shame, and threatened their sexual relationships.	• Fear of losing power • Fear of relational issues • Feeling useless • Stress/anxiety • Disappointment • Shame	“…When I was young my sex life was good…… I could go long sexual rounds with them (women)...as I age my body fails me…my heart still desires…but my body refuses…I am so disappointed in myself as a man… I feel awful… any sensible man must feel awful because that (sexual performance) is the core of manhood …I feel I have lost my manhood... I am disgusted, but what can I do?” (MzeeBwaki)
Confusion regarding the persistence of sexual desire in an aging body	Participants’ accounts revealed a frustrating dilemma: The age-related changes in their body affected their sexual performance, but their sexual desire remained constant.	• Ambiguous feelings about old-age sexuality • Dilemmas related to the persistence of desire • The heart’s desires • Body betrayal • Tension of sexual desire	“…If you manage to have a single round you thank Allah… it is very frustrating… you feel you have lost your image as a man…what hurts most is the fact that the feeling of wanting to have a woman is still there; it is only the body that fails…” (MzeeChande)
Silencing norms associated with old-age sexuality, and the implications on sexual health	Participants said they found it difficult to talk about their sexual problems due to the stigma and shame associated with sexual problems. Majority said they secretly resorted to using either home remedies or traditional herbs.	• Taboo subject • Sexual health concerns • Communication barriers • Shame of admitting failure • Norms of masculinity • Secret strategies • Treatment-seeking barriers	“…the majority of us don’t have the nerve to raise this subject (sexual problems)… You think, ‘How do I start?’ This *puts us in a stressful* situation…” (FGD^a^)

^a^Participant from the focus-group discussions

### “Jando” as a Script for Male Sexuality and Masculinity

Jando is rite of passage for males, which also involves male circumcision. Jando as a male rite provides a cultural script that regulates men’s sexuality. From our data analysis, we discovered that in the cultural context of participants, Jando, or the male initiation rite, serves as a model that regulates and shapes male sexuality and gender-normative practices. Thus, the participants’ ideas regarding masculinity and sexuality should be understood within the Jando framework. MzeeMbonde, 71 years-old from a rural focus group discussion, explained: “…in the past it was a scandal when it was known that a man had not passed through Jando.” MzeeSalumu, 78 years-old from urban group, also commented: “…in the past a man who did not pass through Jando was regarded as an incomplete man.” When asked why was it important for men to pass through Jando rites, the participants claimed that during their youth Jando was the only avenue for men to learn about gender roles and sexual matters, that is, that it was only thorough Jando that a man could acquire the traits that that confirmed his manhood and the attributes of ideal male sexuality.

Generally, the participants claimed that a man who went through Jando training would become sexually knowledgeable. Thus, he would learn, internalize, and endorse the approved sexual norms or scripts that govern his sexuality, including learning about sexual taboos and acquiring essential skills. MzeeSinda, 65 years-old from a rural group, stated: *“*the proper sexual skills could only be acquired through Jando.” This was also discussed by MzeeFadhiri from a rural focus group discussion who said:

…There is no way a young man could get such deep information about life skills, gender roles, and sexuality without passing through Jando training… a lot of teaching happens there [in Jando]… you are taught how to be a man, about your roles …the instruction on sexual matters was a lot (laugh)… yes, because that is the core of manhood... (MzeeFadhiri, 77 years-old)

Through our participants’ narratives we learned that Jando serves a double purpose as a gender script and as a sexual script. Specifically, the Jando model honors two forms of masculinity: the man as breadwinner/material provider and the man as a sexual actor. Participants claimed that a balance between these roles is equated with “ideal masculinity.” As MzeeKibada from an urban focus group clarified:

We learned from Jando the meaning of being a man… we were told that a man who cannot provide for his family and a man who cannot handle a woman well sexually is an incomplete man … a man needs to make a woman respect him, by, for example, going as many rounds as he can [in sexual encounters]… (MzeeKibada, 69 years-old)

From our focus group discussion, it appears that the sexual scripts acquired from Jando include norms that guide behaviour and expectations of male sexuality. The following are examples of sexual scripts mentioned by participants: men’s power lies in performance and potency,” “an ideal man is virile,” “a proper man is good in bed,” “going several sexual rounds makes a man,” “a man with sexual capacity earns respect,” “sexual potency is central to manhood,” “the level of respect a man receives depends on his sexual performance,” and “without good sex, money and wealth cannot satisfy a woman”.

Generally, the sexual scripts participants acquired from Jando in their youth dominate their perceptions, expectations, and experiences of their sexuality in later life. In other words, participants seem to have internalized their perceptions of proper sexual behaviour from Jando. As MzeeMzuzuri, 64 years-old from an urban focus group, insisted: “even if a man provides for his family and gives his woman all the material necessities, if he is not good in bed he is good for nothing, and will not be respected as a man by his woman.” A similar observation was made by another participant from a rural focus group who said: “…a man who cannot handle a woman sexually is an incomplete man … a man must go as many sexual rounds to satisfy a woman …” These extracts (and further examples in Table 2) reaffirmed the notion that a man’s sexuality is based on his performance and that a man’s sexual performance is part of his identity. The older men in our study seem to have internalized this script because they commonly stated that a “real man” is a man who is able to satisfy a woman sexually through good sexual performance.

According to participants, good sexual performance is defined as having prowess, potency, virility, and sexual skills, including the ability to achieve a proper erection and to go several sexual rounds. A common theme was that a man’s sexual capacity or performance is valued above the role of provider. Participants argued that although a man’s masculinity is demonstrated in part by his ability to provide for his family/wife, being a good provider does not give the man power or control over his woman/wife in both the social and the sexual realm if he is sexually weak. The majority of the participants seem to have drawn from the Jando rites in reflecting on their perceptions. For example, MzeeBabu was a widower whose wife had died a year prior the interview. At the time of the interview he had no sexual partner. The first author asked him how he felt as a man about this lack of a partner; he said:

“... (Pause) I think the minute I bring a woman home I would be adding to my burdens... women here expect a lot from men. Now I’m a poor, old man with nothing, and if I bring a wife home, she will demand this and that; she wants food, clothes… You see? But above all she would expect sexual satisfaction…it would be adding more stress and shame…there are changes I see in my body … I doubt if I can make a woman happy…” (MzeeBabu, 68 years-old).

Like many of the other participants in our study, MzeeBabu described the cultural expectations of “a proper man” as requiring him to meet not only his wife’s material desires, but also her sexual desires, as well as that a man has to control his wife’s sexual desires to prove his power over her (i.e., a successful way of being a man). A number of the participants said that a woman who is not satisfied by her husband would either leave him or engage in extramarital affairs. It was also commonly stated that “...if a wife start running around with other men … and the news came out, people will question whether the old man is still able to have sex, and his image will be ruined…” A woman’s lack of satisfaction with her husband’s sexual performance was perceived as a major reason for marital dissatisfaction, and as grounds for divorce. For example, MzeeAli, reported:

… if a man is married to one or more wives and [even if he] provides them with all the necessities, if he fails to satisfy them sexually, they will no longer respect him… they will leave him and badmouth him, saying, “that man is good for nothing.” And people will start speculating that this man has everything, but why does every woman leave him? (MzeeAli, 70 years-old).

To summarize this theme, the scripts on sexuality that the participants acquired from Jando and subsequently internalized apparently dictate their perceptions, guide their sexual relationships and expectations, and influence the meaning they assign to sexuality. Participants stressed that a man has to perform well sexually to satisfy a woman, as well as to control her. In a nutshell, being able to control and please a woman sexually is the most honored way of being a man and thus confirms a man’s masculinity.

### Aging Body and Loss of Sexual Performance

In this theme, participants suggested that body strength is vital for good sexual performance and that a physically weak man cannot perform well sexually. The majority of participants described the difficulties they face in maintaining their sexual performance in old age. A concern that was commonly expressed was that just as their body strength decreases with age, their sexual performance is declining. Commonly participants said that their wives and societal norms set expectations that are quite high. However, most of the participants reported that they have lost their past sexual selves. Instead, they said they rely on “sexual nostalgia,” and they see their youth as a core reference point and ideal for healthy sexuality. MzeeNassoro said:

During my youth I had no impotence problems, my sexual life was good…I used to have several women wherever I went, and I could go long sexual rounds with them... women would praise my sexual performance (nodding his head) ujana ni kama maji ya moto [the literal meaning of this phrase is “youth is like hot water and never fails”]... (MzeeNassoro, 78 years-old)

Aging decline in body functions also manifested in the sexual problems that participants reported experiencing, such as impotence or “uanithi.” In most cases these sexual problems were identified as impotence. This term refers to a range of problems, including the inability to achieve an erection or to engage in several sexual rounds of sex, as well as delayed and premature ejaculation. Our findings indicate that the older men identified three main erection problems: the complete failure to get an erection, the failure to keep an erection for a long time during an encounter, and the ability to get an erection but to be able to go only a single sexual round. MzeeOmary said:

…Once you get old, everything goes against you. You lose all your body strength, and it is like your body betrays you completely.…. I am impotent… this is what disappoints me most… I cannot change anything… If I dare to go against the body's wishes [by going many sexual rounds], the whole body will ache like hell… the body is no longer my ally! The body becomes difficult… no treatment that can cure old age impotence. To be honest, impotence is the greatest annoyance in marital life, and it is even more embarrassing for polygamous men like me… The heart still desires but the body doesn’t allow…it is an embarrassment, for sure. (MzeeOmary, 71 years-old)

A majority of the participants complained that there is a tendency among women, and especially among young women, to expect a man to be a good sexual performer regardless of his age and that this expectation puts them in a difficult situation. Most of the participants expressed concerns about being deemed an “incomplete or good-for-nothing” man who cannot perform well sexually or control his wife’s sexuality. This prospect generated fear, shame, and anxiety in participants.

### Fear, Shame, and Anxiety about Sexuality in Old Age

Fear, shame, and anxiety about sexuality in old age emerged as a prominent theme when participants expressed fear of their inability to fulfil sexual obligations/sexual expectations. A majority of the participants said they believe they had lost power and control over women, which in turn threatened their sexual relationships and elicited in them feelings of shame and distress. Furthermore, because the male sexual script posits that real men should be good sexual performers in order to please a woman sexually and to control her, participants assessed their sexuality based on these expectations. Nearly all participants reported that these expectations were in conflict with both their aging body and their sexual health problems, as well as that this conflict was another source of shame and fear. Generally, the sexual problems related to old age that the participants reported experiencing were perceived as signs of their failure to achieve the ideal of male sexuality, rather than simply as sexual health problems. Thus, these problems represented sources of shame, fear, and anxiety for the men—themes that are captured more richly in the following case examples.

#### Case 1: “I Feel I Have Lost My Manhood.”

In the first of these exemplary cases MzeeMbwiga says:

…the main problem I am facing in my marriage is uanithi [impotence]. I have been facing this problem for almost 5 years now; every day I am worse than the day before. The problem started gradually, but it’s getting worse over time... I do regret it, because I know it is my fault… my sexual behaviour in my youth (having multiple partners) contributed to my current sexual condition… When this problem first came up, I tried many herbs: I drank octopus soap and ate raw nuts and cassava and many other things which I cannot mention here, but the results were not good. I feel I have lost my manhood... I am disgusted, but what can I do? (MzeeMbwiga, 75 years-old)

Participants expressed great frustration with and anxiety about impotence, partly because an impotent male is considered “less of a man or not a man” and partly because impotence is so strongly associated with past sexual experiences. Participants appear to believe that impotence reveals to the current partner that the man was promiscuous or engaged in extramarital sexual activities in the past. For instance, the elements of guilt and self-blame from MzeeMbwiga’s story regarding his impotence are linked to his perception that his impotence is not a health problem, but rather a consequence of his past sexual behaviour. Moreover, impotence is associated with infertility, and if a man is unable to father a child, he is seen as having wasted his life. This anxiety was especially acute among participants who had a young wife or were polygamous. Apart from their fear of losing their power, their fear of losing their partner (loneliness) stems from the fact that these men depended entirely on their wife or wives for their daily, as well as for their long-term, care. Thus, the fear of being left without care heightened their distress.

#### Case 2: “My Sexual Performance Ceased Completely; I Feel Awful.”

MzeeMdundo now lives with a new wife, almost half his age. This is his third marriage after his previous two wives passed away. He married her 2 years ago. He said:

last year I found out I had heart problems and a few months later, I was found to be diabetic… when I started diabetes treatments things turned worse… my sexual organs became numb. I thought maybe this was because of the medication, but 3 months ago I stopped taking the medication, thinking maybe things would improve, but my sexual performance ceased completely…I feel awful…My wife seems to be concerned and supportive, but I am not sure if she is for real…I don’t know. Maybe one day she will leave me and find someone who is younger…

Initially, focus-group participants had said that their fear and anxiety was associated with having engaged in extramarital affairs. However, in the in-depth interviews, the majority of the participants—and especially those with a younger wife or a big age gap between themselves and their partner(s) (including those in polygamous marriages)—revealed that they were embarrassed by their performance issues within their marriage. Men’s superior power and dominance are embedded in characteristics such as having sexual prowess and virility, having sexual skills, being able to achieve an erection, and being able to go several sexual rounds. The difficulties the men reported facing in meeting these expectations as they grew older was identified as a source of anxiety. Very few participants said that they are not worried about sexual difficulties and said that they accepted it to be a normal aging process, for example MzeeKyondo, 71 years said: “…I don’t feel less a man because of impotence, I had enough of it …I thank God for granting me many years and I don’t complain because I am still breathing…”.

### Confusion Regarding the Persistence of Sexual Desire in an Aging Body

In the participants’ narratives, a common thread emerges of the persistence of sexual desire in an aging body. The majority of participants claimed that aging affected the functionality of their body, but not their sexual desires. As MzeeIbrahim, 81 years-old, commented: “…any man, even if he is 100 years or more, would have an appetite (desire) for sex.” The discrepancy between the functionality of the body and sexual desires was perceived to being a source of pain, frustration, and shame for a man because it undermines his sense of masculinity. Participants expressed confusion regarding the inability of their aging body to respond to their persistent sexual desires. For example, MzeeAmani expressed frustration with the decline in his sexual performance. He described his efforts to act on his desire as causing him anxiety. He said his ego is threatened when he failed after several attempts and that he felt ashamed. He put the blame on his body; he said “…It is so frustrating; the feelings are there… I feel helpless and become angry at my own body… I feel ashamed and worry about what she thinks of me…. sometimes I ask myself, why I attempted it?” (MzeeAmani, 74 years-old).

The previous quotes powerfully encapsulate the participants’ divergent attitudes toward sexuality later in life. Their frustration is made clear in MzeeChande’s account (see Table 2); the internal struggle between what he desires and what his body can do reveals his dilemma. This situation (in which sexual desires coexist with the aging body) was referred to by participants as a “body betrayal” and was perceived as being a painful, frustrating, and shameful experience because it undermines their sense of masculinity and sexual-being.

### Silencing Norms of Old-age Sexuality and Sexual Health

The last theme that emerged from our analysis is about the silencing norms associated with male sexuality. The cultural and the societal norms related to masculinity were raised in focus group discussions. A primary constraint faced by older men is a lack of space to discuss their sexual problems because of the silencing norms regarding old-age sexuality. These norms discourage men from asking or talking about their sexual weakness/problems. Thus, a man is encouraged to conceal his sexual weaknesses to protect his image as a powerful and proper man. These silencing norms are reflected in sayings such as “Mwanaume lazima uwe na kifua” [A man needs to keep his secret in his chest] and “mwanaume alipewa Kikoromeo kwaajili ya kutunza siri” [A man’s Adam’s apple is for keeping secrets]. Furthermore, participants revealed that women do not believe that men age sexually as women do, a perception that is reflected in the saying “Ng’ombe azeeki maini” (A cow liver does not age).

These attitudes seem to have constrained older men from sharing their sexual problems with their sexual partners, peers, and professionals. Moreover, these norms deny older men the space to express their fears and anxieties because such behaviour is perceived as womanly and would call their manhood into question. MzeeOmbeni, 67, a participant from a rural focus group elaborates by pointing out that a majority of men do not have the nerve to share their sexual problems to their partners. Commonly, older men said they feel that the disclosure of their sexual problems to their wife could create unnecessary suspicions of infidelity. As MzeeChilingi, 69 years, commented: “…once you have told your wife about it she will become suspicious… She may feel too shy to ask you openly, but you know she has questions in her head…to preserve your image you better avoid talking about it …”.

Furthermore, according to participants, an older man never discloses his problems if he wants to preserve his image as a sexually powerful man. A majority of participants, especially those who were married to a young woman, revealed that they had used secret strategies to regain their sexual performance. On the other hand, participants’ claim that women do not realize their age-related decline in performance could partly be contributed by the silence norms: Most of these men have not openly discussed their sexual well-being with their partners and thus are not aware of its actual impact on their partner’s age-related decline in performance. MzeeAyubu 72 was among the few participants in our study who broke the silence norms and talked to his wife. Mzee Ayubu said;

…it is very difficult to start this talk…after avoiding sex several times….it is good she asked me and then I got courage to tell her that I am facing this problem (impotence)…she understood me because we have been together since we were younger… some women would conclude without asking that lead into marital conflict. (Mzee Ayubu, 68 years-old)

Generally, most participants agreed that it is important for a man to maintain his image among his partner, and friends and that he should never disclose his sexual problems. The lack of space to communicate their sexual problems and to express their fears due to the pressure to conform to norms of masculinity seem to have resulted in poor sexual health-seeking behaviour among older men. This may also mean that older men feel unable to disclose their sexual problems or seek treatment even when they have contracted a sexually transmitted disease. For instance, the findings of our study show that because of fears associated with silenced sexuality, many men use traditional herbs and home remedies to treat sexual problems rather than seeking out professional health care. The comment from MzeeKulwa, 74 years-old, from rural group further illustrates “…I don’t know which herbs will help me regain my performance…I have been struggling with different herbs, but none of them have helped me regain my manhood.” MzeeSamweli, 71 years-old, a participant from an urban focus group said: “I know some won’t say it, but old men here are struggling…trying different herbs to conquer their impotence …very few succeed… our bodies are already weak… nothing will help us.”

Commonly participants said they believe that sexual problems such as impotence in old age and diseases that are associated with male reproductive organs such as the “tezi dume” [prostate gland] are caused by the individual’s past sexual behaviours or experiences (such as having had multiple partners in his youth). As MzeeKitwana, 66 years from a focus group, commented: “….those who were promiscuous in their youth have these [prostate gland illnesses].” In such situations, (due to the stigma attached to these conditions), men tend to resort to either home remedies or to traditional herbs, although the appropriateness and effectiveness of these approaches is often unclear.

Moreover, FGD participants pointed out that due to the pressure of having to conform to dominant forms of masculinity, many men suffer from health problems and even death. Although there is no direct evidence of suicide in our study, group participants from the urban areas reported that there had recently been some suicides via hanging: “Some because they perceived themselves as failures; others because they didn’t have support…Others had maybe lost their hope and their dignity. So they think it is better to die quickly than to die slowly!” Participants also articulated alternative strategies to cover up their sexual weaknesses or to try to conform to dominant male sexuality norms to avoid embarrassment and shame. These strategies ranged from denial to displacement, but also included acceptance. The common response described by the men was to use traditional herbs or foods, which are said to boost sexual performance (see case study 1). Some said they came home late in order to avoid sex and the “shame of failing to perform”; others said they turned to alcohol, and still others said they became rude and violent. A few indicated that they simply accepted their sexual decline as part of the aging process. Because of these silencing strategies, participants felt confused about what they should do, and they lacked adequate space to communicate their sexual problems.

## Discussion

The present study was undertaken mainly to examine older men’s perceptions of sexuality in relation to the norms of masculinity, that is, the masculine sexual script. Analysis of the individual interviews and of the focus group discussions together produced the overarching themes regarding the meaning older men assign to their sexual experiences in relation to cultural norms of masculinity (see Table 2). Our findings show that Jando serves as a model and a script for male sexuality that outlines the expectations and rewards of male sexuality. Or, to put it another way, these scripts are descriptive of shared expectations, and they reflect cultural norms and values as they are perceived and internalized by men. Within this script, being seen as a good sexual performer preserves a man’s image and enables him to fit into normative masculine sexuality scripts.

Consistent with previous research (Masters et al. 2013; Maticka-Tyndale et al. 2005; Stephens and Eaton 2014), our results show that the scripts older men internalized (from Jando in their adolescence) have become tools that help them make sense of their sexual experiences, shape their perceptions, and guide their sexual behaviours. For instance, these men have fairly masculine sexual scripts that equate good sex with phallocentric sexual performance, and they believe that by living up to this ideal, they are “doing gender” in a stereotypical way (West and Zimmerman 1987). Thus, our findings are in line with those of Crowell (2011) and Masters et al. (2013) who maintained that sexual scripts for male sexual behaviour are reduced to sexual performance metrics such as penetration, achieving erection, and going several sexual rounds/demonstrating sexual stamina. Through sexual performance men realize their cultural ideal of masculinity (Smiler 2006). Yet for the majority of the men in our study, their efforts to adhere to the scripts of normative masculine sexuality into old age generated feelings of fear, anxiety, and distress—ultimately engendered feelings of low self-esteem. These findings confirm the results of previous studies (Mahalik et al. 2007 and Scott et al. 2013) which showed that living up to a masculine sexual ideal can lead men to feel distress, anxiety, and fear. Likewise, our findings are in line with those of Morrell (2005) and Mahalik et al. (2007) who observed that traditional masculine gender norms play a critical role in men’s mental health and well-being in later life.

Our results go one step further by showing that the fear, anxiety, and distress participants reported are linked to the aging-related body limitations that affect men’s ability to endorse normative masculine sexuality scripts. Specifically, the fear of losing power and control over women, and therefore of a loss of status, leads to distress. These findings resonate with the argument put forward by Fracher and Kimmel (1995) that sexuality is a site for experiences of power. Thus, the lack of potency is seen as a failure to live up to the masculine sexual ideal and is likely to threaten a man’s self-esteem These findings are also in line with Charmaz’s (1995, p. 658) argument that “aging reduces a man’s status in masculine hierarchies; shifts power relations and raise self-doubt about masculinity.”

In addition, the older men laid the blame for their failures on their bodies; “the heart desires but the body refuses” was a common expression of this experience. Older men’s perceptions of their aging bodies support the views of feminist gerontologists. For example, Tulle put forward the notion of “disruptive bodies” (Tulle 2015, p.127), whereas Calasanti (2010, p. 723) referred to these processes as the “degendering” or the devaluation of late life identities. Our findings suggest that the devaluation of men’s masculinity in later life is largely a result of their inability to endorse normative masculine sexuality scripts (due to bodily limitations) and that there are thus discrepancies between an individual’s sexual experiences in later life and the cultural scripts of masculine sexuality. These discrepancies affect older men’s processes of self-identification (such as the feeling of being an incomplete or a lesser man) to the point of generating anxiety, as well as to interfering with their process of self-identification (Antoninetti and Garrett 2012).

The alienation of the body from the self was also reflected in the work of Featherstone and Hepworth (2009) who found that older people compared the aging experience to wearing a mask. We can link Featherstone and Hepworth’s concept of the body’s mask with participants’ statements regarding the concept of “body betrayal.” Body betrayal was a term participants used to describe the conflict that arose when their sexual desire (appetite for sex) persisted, but aging made it harder for them to perform sexually. This gap between desire and performance was perceived as being the main source of a man’s frustration, shame, and distress because it undermined his sense of masculinity. In this respect our results were consistent with those of previous reports from Malawi by Freeman and Coast (2014), as well as with Sandberg’s (2013) argument that men’s sexual desire is natural and persists across the life course. These findings are also congruent with Gott and Hinchliff’s (2003) observation that although older men experience sexual difficulties, they are not asexual and that they continue to have sexual feelings and desires.

Our findings call into question Morrell ‘s (2005) claim that when men are in crisis, they become eager to rethink their masculinities and to adapt to new ideas with respect to masculinity, as well as Gott and Hinchliff’s (2003) suggestions that when older people experience sexual problems they reprioritize the role of sex in their life. Nearly all the men in our study expressed a desire to conform to the sexual script of male sexuality (and when they failed to do so they utilized a variety of strategies to cover up their sexual weaknesses). Even the men who had opted to avoid sex did so to cover up their sexual weaknesses and were not rethinking their masculinity or adopting new ideas. We argue that this shows that the desire among the older men to conform to the script seems to be linked to power relations (i.e., to a desire to maintain their position in the gender hierarchy). Because sexuality is, arguably, a site for experiences of power, a lack of potency represents a failure to live up to the masculine sexual ideal and is therefore likely to threaten a man’s self-esteem (Fracher and Kimmel’s 1995.

Lastly, most of the men in our study had not discussed their sexual difficulties with their partners or with their health practitioners due to existing male sexual scripts that constrain men from sharing their sexual problems in order to preserve their image as “proper men.” These silencing norms not only estrange men from their own sense of self and affect their sexual agency, but also seem to lead older men to engage in poor health-seeking behaviours, such as using traditional—and largely ineffective—remedies such as herbs. It therefore seems that the decision to conceal their sexual problems, and thus to avoid seeking treatment for their sexual problems from professionals, is interpreted as a sign of possessing a masculine self. These findings are consistent with those of studies such as Marcos et al. (2015) that identified poor health-seeking behaviours as ways to construct masculinity, even though they are detrimental to men’s health. Likewise, our findings support the view expressed by Connell (2005) that the pursuit of hegemonic masculinity through normative masculine sexuality scripts comes at the expense of men’s health.

### Limitations, and Future Research

Despite the strengths of the current study, which include making the experiences of marginalized populations’ visible and scrutinizing how the masculine sexual script affects older men’s perceptions of their sexuality in later life and has detrimental effects on their well-being, it has some limitations. Our findings are derived from older men who largely share cultural backgrounds, and many are polygamous. It is likely that having more than one wife (or younger wives) may cause older men to perceive their sexuality the way they did and that the influences of polygamy overshadow the influences of masculine sexual script. Future research should explore perceptions of sexuality among men who differ in terms of cultural background and marital status.

### Practice Implications

Our findings indicate that adhering to a masculine sexual script affects older men’s perceptions of their sexuality in later life and have detrimental effects on their well-being. The sexual scripts drawn from participants allow for the particular role culture plays in shaping health behaviours. Based on our findings, we argue that older men may benefit from age-related interventions tailored to their cultural background. For example, awareness of the prevailing sexual script points where alternative directions can be taken and provides insights into potential strategies for developing sexual-health interventions. Experience has shown that programmes and interventions which are not cultural sensitive and age-friendly are likely to fail (WHO, 2010). Thus placing such interventions within the context of existing scripts may support older men in developing new normative patterns of sexual scripts, which in turn may enhance their emotional well-being.

Some of these interventions may require trained healthcare providers on mental health issues to bridge the gap between the internalized scripts of ideal male sexuality and the reality of aging. Furthermore, the findings of our study show that sexual silence is a cultural value that is deeply woven into masculine sexual scripts. These silencing norms seem to be key determinants of older men’s sexual behaviour; for example, our findings indicated that majority of older men were not able to discuss sexual problems with their partners or practitioners and often resorted to using unhealthy alternatives to avoid shame and stigma. Such silence potentially creates a barrier to older men from seeking help for reproductive healthcare. Our study calls for a gender transformative interventions that raise awareness about unhealthy masculine norms. These interventions should empower men to resist stereotypes and limitations imposed by masculinity scripts.

Additionally, although many sexual-health interventions demonstrated considerable attention to reproductive and sexual health of younger people, the findings of our study can be used to tailor interventions to the unique sexual health-promotion needs of older men, who traditionally have been overlooked and underserved by public health interventions. Thus, this information opens the door for the development of age-gender appropriate and effective sexual health empowerment programs seeking to meet the needs of older men. Besides, population aging and the rollout of antiretroviral therapy (ART) in Tanzania necessitate a re-examination of many ageist stereotypes and youth-based sexual health interventions. Ageist stereotypes and lack of sexual health programs for older people may increase the risk of HIV/AIDS among this group.

### Conclusion

A major concern for feminist gerontologists is to bring together socio-cultural dimensions in order to understand the aging process. In our study, we attempted to respond to this call by revealing how cultural expectations of masculine sexuality (sexual scripts) shape the way older men perceive their masculinity and the impact these perceptions have on their sexual and mental health. Thus, our findings represent an important contribution to the ongoing discourses of “aged by culture.” Arguably, the ways in which older men respond to sexual changes and how they integrate these changes into their well-being are embedded within a culture framework (Minichiello et al. 2005). We hope with the present study to contribute understandings of sexuality as a cultural construct (internalized) and to highlight the ways that this internalized masculine sexual script further cause distress for men in later life, ultimately, affecting their well-being.

## Electronic supplementary material

ESM 1 (DOCX 22 kb)
