# Supplementary material for: “The Heart Desires but the Body Refuses”: Sexual Scripts, Older Men’s Perceptions of Sexuality, and Implications for Their Mental and Sexual Health
Source: Sex Roles. 2017 Sep 9;78(9):653–68. doi: 10.1007/s11199-017-0822-3 (PMC5897462; doi:10.1007/s11199-017-0822-3)
Supplement: Supplementary file 1 — (DOCX 22 kb) [file 11199_2017_822_MOESM1_ESM.docx]

Online supplement for Rutagumirwa, S. K., & Bailey, A. (2017). “The heart desires but the body refuses”: Sexual scripts, older men’s percpetions of sexuality, and implications for their mental health. *Sex Roles*. Sylivia Karen Rutagumirwa, University of Groningen. Email: [s.k.rutagumirwa@rug.nl](mailto:s.k.rutagumirwa@rug.nl)

**Guide Used for Focus Group Discussions**

***Part I: Masculinities and*** *J****ando socialization***

1. What is the number one thing that men are judged on in your society
2. What does masculinity mean to you?

**Probe**: What are the traditional masculine ideologies in your community?

**Probe**: for physical, sexual expectations, emotional, social)

1. How are men socialized into masculinity and sexual relationships (thorough Jando)?

**Probe**: What norms relating to sexuality are communicated to men in Jando rituals?

1. What are the goals of the traditional teachings?

**Probe:** Jando influences on ‘being a man’

**Probe**: Jando influence on sexual behaviours

**Probes**: Others sources of influence

1. How do Jando influence the ways in which older men perceive and feel on their sexualities?
2. What are the norms of male sexuality you were told from Jando?

***Part II: Shared norms on male Sexual and relationships***

1. What kind of things you were told about male sexuality from Jando?

**Probes**: norms that guide behaviour and expectations of male sexuality

1. According to your socialization experience, what does a sexually responsible man act like? **Probes**: norms or scripts

***Part* *III*: Main sources of information**

1. Do men of your age talk openly to (partners, other people, friends and professionals) about sexual and related issues including sexual problems? How? - Joke, serious, giggle, give advice, swap information etc

**Probes**: (If no) why?

**Probes**: Norms on male sexuality and masculinity

1. Whom or what do older men rely on for sexual information?
2. What do older men perceive to be the major challenge to conform to norms of masculinity in late life? How do they deal with those challenges?

**Probe**: coping strategies

**Guide Used for Individual Interviews**

**Section A: Background characteristics:**

No. of interview __________________ Name__________________

Date__________________

Start __________________End__________________

Age__________________Tribe__________________

Location__________________Religion__________________

1. Have you been to school? If yes, up to which level? __________________
2. What is your source of income? /occupation? ________________
3. Are you married? ___________________________
4. (if yes) how many wives_____________________
5. What is your spouse’s age ____________________
6. How many children do you have? _______________________

**Section B: Opening questions**

1. What is the number one thing that men are judged on in your society*?”*
2. What does masculinity mean to you?

**Probe**: for physical, sexual expectations, emotional, social)

**Probe**: sources of sexual and influence

1. Do you perceive yourself as masculine?

**Probe**: Changes in self-perceptions

1. Has your view of your own personal masculine identity changed as you have become older?

**Probe:** why,

**Probe:** compare to when you were younger

**Section C: Cultural sources of male socialization on sexuality**

Now I’m going to ask you some questions about your experiences with sex socialization, it may have been awhile so I don’t expect you to remember every detail. I just want to know what you remember from that time in your life.

1. When and in which ways were you aware of your sexuality?

**Probe**: Jando as source of sexual socialization

**Probe**: Who taught it?

1. What kind of things you were told about male sexuality from Jando?

**Probe:** behaviour, roles, responsibilities, dos and don’ts:

1. What did you think about it; in which ways they influenced the way you perceive and experience your sexuality

**Probe:** experienced, and defined your sexuality?

**Probe:** What of this information did you feel like was most important to you personally?

1. Which messages were taught to you about sexuality from other sources?

**Probe:** e,g, school ,religious messages

**Probe**: In which ways they influenced the way you learned, experienced, and defined your sexuality?

**Section D: Sexuality and Sexual behaviour and masculinity in later life**

1. Can you please tell me about your personal history with regard to sexual life?

**Probe**: Past and present sex life

**Probe:** feelings about sex, level of satisfaction, disappointments?

1. Do you think what you were told/ learned about male sexuality (from Jando or other sources) shape/affect the way you perceive your male sexuality now?

**Probe:** If so, how?

**Probe:** Past and present sex life? How?

1. What changes in sexual experiences/behaviour do your experience as you age?

**Probe:** ability, affection, sexual desire, sexual arousal, erections, orgasmic consistency, sexual satisfaction)

**Probe:** Experiences of each of these changes, including their emotional impact?

**Probe:** Does masculinity play a role? How?

1. What are your perceptions of the age related change in sexual functioning on your masculinity/manhood?)

**Probe:** Masculinity; power relations masculinity norms and pressure

1. Does age related changes in sexuality (e.g sexual functioning) affect your view of your own personal masculine identity?

**Probe**: How and why?

1. What do you perceive to be the major challenge to conform to norms of masculine sexuality in late life? How do they deal with those challenges?

**Probe**: sexual problems; communication

**Probe:** coping strategies

**Section E: Aging, body and Sexualities**

1. Do you perceive your body as less functional (declining) now that you are getting old?

**Probe**: If (yes) how did that make you feel?

1. Are you able to do the same things with your body now compared to when you were younger?

**Probe:** sexual activities

1. How does that make you feel—particularly in terms of masculinity? Do you think ideal male sexuality matters to someone in very frail older age?

**Probe:** If so, how and why?

**Probe**: If not, why not?
